# Cow blood – A superior storage option in forensics?

**DOI:** 10.1016/j.heliyon.2023.e14296

**Published:** 2023-03-08

**Authors:** Ursula Windberger, Andreas Sparer, Johann Huber

**Affiliations:** a Decentralized Biomedical Facilities, Core Facility Laboratory Animal Breeding and Husbandry, Medical University, Vienna, Austria; b Vetfarm Kremesberg and Clinic for Ruminants, Section for Herd Health Management, Veterinary University, Vienna, Austria

**Keywords:** Cow blood ageing, Bloodstain pattern analysis, Blood storage, Rheology, Hematology, Drip pattern

## Abstract

Given the use of modified blood products (e.g. leucocyte depleted erythrocyte concentrates in SAG-mannitol, dehydrated blood powder, defibrinated blood), drawing blood from conscious animals while minimizing their stress is a good option to obtain blood for bloodstain pattern analysis. Nevertheless, the blood must be well described since individual differences in quality can occur, and storage will influence blood components qualitatively and quantitatively. Cow has been discussed as a suitable source of blood supply, but current data lack hematological and full rheological perspectives. This project includes the respective parameters in combination with passive drip pattern experiments during refrigerated storage in multiple study arms. Cow blood displayed a constant increase in viscosity (at high shear rate: 1000s^−1^), reflecting the expected reduction in red blood cell (RBC) flexibility. RBCs shrank but remained intact with very few irregular shapes, therefore there was no evidence of hemolysis. Influence of storage on stain size in passive drip pattern experiments with different substrates was minimal. However in cows, it is not hemolysis but an early change in suspension properties that indicates storage lesion. Viscosity (at low shear rate: 1s^−1^) of some blood samples increased three-fold (peaking at day 14), transitioning sharply to near-Newtonian (almost shear-independent) behavior thereafter. The higher this increase in viscosity, the greater the increase in the number of satellite spatter on glass. In order to ensure high quality simulations in the future, comprehensive rheological analyses to detect gradual changes in blood pseudoplasticity should be implemented in the forensic discipline of bloodstain pattern analysis.

## Highlights

• Bovine RBCs shrink but remain intact during four weeks of cold storage.
• Influence of storage on stain size of passive drip pattern experiments is minimal.
• Storage lesion cannot be detected by hemolysis but by blood behavior at low flow.
• End point of storage is the switch towards Newtonian behavior starting at day 14.
• The greater the change in suspension property, the higher the rise in satellites.

## 1 Introduction

The use of animal blood in the analysis of fluid dynamics and spatter creation for forensic applications can only be a compromise, just as in biomedical research animal experiments are only models for pathophysiological processes. Nevertheless, limited availability of human blood samples for simulations, frequent staff training and innovation research creates the need for alternatives, and tempts even the use of modified blood products (leucocyte depleted erythrocyte concentrates in SAG-mannitol, defibrinated blood, resuspended blood powder). With regard to the use of artificial materials, preference is certainly given to native blood, even if it is animal blood, but it is crucial at the same time that the used blood has good quality. It is also important to know the differences between the chosen animal blood and human blood that affect the test results. Animal blood differs species-specifically from human blood concerning its mechanical behavior; e.g., whole horse blood is inappropriate to simulate human blood and should be generally avoided, and sheep and pig blood compares to human blood only at distinct shear rate ranges [1]. This is due to the different nature of red blood cells (RBC) between species and the quality of their embedding in plasma [2], creating a species-specific blood viscosity [3,4] and, more generally speaking, behavior in the shear fields [5]. If the animal blood of choice must be stored prior to its use, further uncertainties will appear depending on the storage conditions. Therefore, the effects of storage must be known for each type of blood suspension whose use is considered.

Flow resistance of blood suspensions measured by viscosity is a function of shear rate (shear viscosity) but can also be a function of an extensional force (elongational/extensional viscosity). In real scenes several nonsteady and kinematically mixed flows will occur and shear flow describes only one part of the shape change that a shed blood volume undergoes outside the body. Interfacial conditions like surface wettability and porosity complicate matters. Whatever is the flow field, one might think that especially high flow rates would matter in forensics. This is due to the fact that impacts occurring at crime scenes are mostly high - e.g. when firearms are discharged. At first sight, an animal species will be sought whose blood behaves similarly to human blood when subjected to high stress inputs. Pig is a good choice here, as it offers similar RBC deformability to human, resulting in a good match in high shear viscosity at equal hematocrit (HCT), and hence a good match in blood behavior at high impact [1]. However, porcine blood forms clusters with storage duration so that the suspension loses its bulk cohesion, which changes rheology completely. This was reflected in a smaller stain size in previous passive drip simulations [6]. In addition to cluster formation, good quality pig blood may be difficult to obtain in slaughterhouses. We believe that when bloodletting of live animals is not possible, slaughterhouse collections must lie in the hands of trained personnel using standardized protocols [7] to ensure not only a collection of pure blood without interstitial fluid and a correct mixing with the anticoagulant, but also a selection of unstressed animals. Slaughtering is usually time-consuming and associated with great emotional burdens. In this context, one has to ask oneself to what extent stress-free blood sampling in a slaughterhouse is even possible. Drawing blood from conscious animals in their usual environment is certainly superior as it avoids the stress of transport [8] and crowding in front of the site of slaughter, and it allows blood collection with minimal suction [6]. The nervous character of pigs make the decision to prefer cows easy. Blood from cows offers the advantage that large quantities can be drawn with short restraint of the animal and that RBC aggregation is low [4]. Problems caused by RBC clustering during storage thus may be delayed. These considerations prompted us to turn our interest to cow blood.

We are not the first to have studied the storability of cow blood with a forensic focus. In a recent study [9] it was shown that blood properties change quickly (within few days) if cow blood is stored at 22 °C. As this time interval might be too short for some users, we investigated to what extent it can be prolonged if CPDA-1 solution (the gold standard for whole blood preservation in human blood donations) and a storage temperature of 4 °C are applied, and if samples are agitated periodically during the weeks of storage to resuspend accumulated blood cells.

## 2 Materials and methods

### 2.1 Blood sampling

Blood was drawn from 11 clinically healthy Simmental cows (age: 5.5 ± 2.4 years, housed at Vetfarm Kremesberg, Veterinary University Vienna, Austria), by puncturing the jugular vein. 50 mL syringes were prepared as described recently [6]. Briefly, CPDA-1 was extracted from standard CPDA-1 blood bags (Fresenius Kabi, Germany) and inserted into perfusor syringes (Braun, Germany; 7 mL of CPDA-1 + 43 mL of blood, resulting in a 1:7 dilution of blood). The syringes were connected to short IV lines attached to sterile needles. All air was removed from these systems prior to punctuation of the vein. Withdrawal of cow blood was approved by the institutional ethics and animal welfare committee and the national authority according to §§ 26ff. of Animal Experiments Act, Tierversuchsgesetz 2012 - TVG 2012, reference number BMBWF-68.205/0092-V/3b/2019. On the way to the lab (approximately 90 min), blood samples were kept at room temperature in an insulated bag. Once in the lab, samples were analyzed immediately in study part 1, centrifuged at 2000g for 15 min to adjust the hematocrit (HCT) to a physiological bovine value in study part 2, and centrifuged at 2000g for 15 min to adjust the HCT to the human standard in study part 3. After having finalized the first set of measurements on day 0 in each study part, we stored the samples at 4 °C in 50 mL syringes (by using new 50 mL syringes in part 2 and 3). Prior to each analysis and also once between the different time points in study part 2 and 3 the syringes were placed in an overhead agitator (REAX2, Heidolph, Germany) at the lowest available speed for 10 min to allow re-suspension of blood cells.

### 2.2 Study parts

The study comprised three parts. Study part 1 used native blood of six cows (A – F) that was diluted by CPDA-1 solution (HCT: 23 ± 2%). This study part was validated four months later by using the same animals (study part 2). Two of these individuals were no longer available, therefore only four blood samples were tested (A,B,D,E). To compensate for the dilution by CPDA-1, a small plasma volume was removed, thereby restoring the HCT to physiological bovine values (27 ± 1%). In study part 3 the HCT of blood samples from a new group of five cows (G – K) from the same supplier was elevated to a human standard (38 ± 1%). All study parts comprised periodic lab tests with standard hemograms, rheometry, and passive drip pattern simulations, albeit at different time intervals.

### 2.3 Hemograms

Hematology was performed using an ADVIA 2120i system (Siemens, Germany) that applies photometry, flow cytometry and impedance measurement. To observe the onset of hemolysis, aliquots of the blood samples were centrifuged using a Haematokrit 2010 centrifuge (Hettich, Germany). A change in the colour of blood plasma in the glass capillaries was assessed visually and the capillaries were photographed. RBC shape was assessed microscopically in bright field at days 0 and 28, resp. 35 (Olympus IMT-2 mounted to a Nikon DS-Fi1 camera/DS-U3 digital sight tool, Japan).

### 2.4 Rheometry

Physica MCR 301 and 302 rheometers (Anton Paar, Austria) equipped with a Peltier controlled stainless steel cone-plate system (sand-blasted cone with diameter: 50 mm, angle: 0.992°, truncation: 0.100 mm, sample volume: 0.59 mL) were used. The test system was covered with a tempered hood equipped with an evaporation blocker to avoid sample drying. Rheological tests were performed at 22 °C to resemble ambient temperatures that might often prevail in BPA experiments. Tests were run at simple and oscillating shear flow. An overview of the parameters obtained is given in Table 1.

**Table 1:** Rheological parameters tested.

Parameter	Definition	Macroscopic appearance
shear viscosity (η) at 1 s^−1^ [mPa*s]	Flow resistance at low rotation	RBCs start to tumble and roll, and RBC cluster flow slowly and eventually align normally to the flow direction
shear viscosity (η) at 1000 s^−1^ [mPa*s]	Flow resistance at high rotation	Singular suspended RBCs elongate in the flow direction. If cells can align in the streamlines, viscosity is reduced
shear-thinning	low shear vs. high shear viscosity ratio	Degree of dynamic changes associated with the variation of the rotation speed
Elastic shear modulus (G′) (in mPa)	Resistance to sinusoidal oscillation. If structures are present in blood that can store the deformation, the phase shift between the input and the output signals are phase-shifted less than *Δφ<<π/4*. In such case the material shows solid-type behavior. If the phase shift is larger than *Δφ>π/4,* viscous behavior dominates, and at *Δφ =π/2*, the material shows only viscous behavior.	The deformations of the cone of the test system are transmitted to the sample and the structures in blood can uptake this deformation and guide them to the plate on the opposite side.
Viscous shear modulus (G'′) (in mPa)		The amount of stress input that cannot be stored is used for flow (e.g. gliding of components along each other, breaking hydrogen bonds, friction).
Loss factor (tanD) (dimensionless)	tanD = G'′/G	The lower is tanD, the more elastic (= gel-like) is blood.

#### 2.4.1 Simple shear flow

To obtain shear viscosity (η) we created isothermal shear strain-controlled flow curves (1000 - 1 s^−1^) based on a logarithmic shear rate ramp. The software calculates viscosity from the shear stress versus shear strain relationship (η [Pa * s] = τ [Pa]/$\dot{\gamma }$ [s^−1^]). Shear thinning was calculated as η_1s-1_/η_1000s-1_.

#### 2.4.2 Sinusoidal shear flow

New portions of the blood samples were sheared in an oscillatory shear field by controlling the shear stress amplitude (A). Frequency sweep tests were run in linear mode (at 10 mPa amplitude) at logarithmic frequency ramp (0.1–3.16 Hz). Here the output signal is the resulting sinusoidal shear deformation of blood. The stress-strain relationship at frequency (ω) identifies the dynamic shear modulus (G* = τ (ω,A)/γ (ω,A)). To separate between the elastic (G′) and the viscous (G'′) modulus, G* is multiplied with cos(δ) for determination of G′, and with sin(δ) for the determination of G'′. The phase shift angle (δ) is the lag phase between the applied shear stress and the resulting shear strain, and it indicates the in-phase and out-of-phase components of G*. From the frequency spectrum we extracted the loss factor (tanD) at 0.8 Hz, as this resembles the physiological heart rate of cows. TanD displays the relative proportion of the two shear moduli (tanD = G'′/G′).

### 2.5 Drip pattern simulations

Blood was dripped in a 90-degree angle from an intravenous line (nozzle size: 3 mm) on the substrate (distance between outlet and contact surface: 50 cm) using a Combimat 2000 perfusor pump (Braun, Germany) set to a velocity of 15 mL per hour. First drops were always discarded to guarantee constant blood flow in the line. We did not install windscreens on the sides, but experiments were carried out in a closed room, where air movement was limited to an absolute minimum. Drip patterns were created in technical triplicates on a glass plate with a template having black concentric circles underneath and on white cardstock paper (200 g/m^2^, Max Bringmann KG – folia, Germany, only in study parts 2 and 3).

On glass, two consecutive blood drops were dripped and photographed (EOS 1100D camera (Canon, Japan), EF-S 18–55 mm objective (Canon, Japan), ISO: 400, white balance: 7000 K). For photography, the scene was illuminated with 5500 K fluorescent lamps in support of a standard ceiling light with neon tubes. The number of satellite spatter around the parent stain was counted within a radius of 7 cm around the point of impact (merged stains were registered as one). The size of the parent stain was determined visually between the concentric circles (5 mm distance) of the underlying template. In study part 3 videos were recorded at days 0 and 28 to show the backsplash of blood when the second drop impacted the first drop (Mavo Edge camera (Kinefinity, China), 18–35 mm CINE T2.0 objective (Sigma, Germany), aperture: 4.0, ISO: 2560 (native), colour profile: log, 280 fps, shutter speed: 90°, resolution: 2048 × 800, crop factor: super16, white balance: 5600 K). For filming, the scene was illuminated with a 4100 K LED station in addition to the neon tubes. Video 1 was subjected to a speed reduction by 50%.

On cardstock paper only one drop was dripped. To evaluate the size of the stain, a grid with black lines (2 mm line spacing) was printed on a standard overhead transparency, aligned, and fixed on the paper. Thereafter the stain plus the overlaying grid were scanned (DCP-L6600DW (Brother, Japan; resolution: 600 dpi) and the resulting image was assessed visually.

In study part 1, we additionally tilted the glass plate at a 45-degree angle and dripped a single drop onto this surface from a height of 10 cm by using our perfusor system. A stopwatch was used to measure the time until the blood flow created by gravity reached 10 and 20 cm marks. Mean values of technical triplicates were used for calculations.

### 2.6 Data processing and statistics

We used following software programs for data aquisition, analysis, and illustration: RheoCompass (version 1.22; Anton Paar, Austria), Excel (version 2016; Microsoft, USA), GraphPad Prism (version 6; GraphPad, USA), NIS-Elements (version D 4.13.5; Nikon, Japan), GIMP (version 2.10.30; The GIMP Team, USA), Premiere Pro CC 2022 (version 22.1.2, Adobe, USA), and InDesign CC 2022 (version 17.0; Adobe, USA). One-way ANOVA (including Geissler-Greenhouse correction) was performed to see whether parameters changed during storage. The Pearson's correlation coefficient was calculated to determine relationships between parameters. Deviations from mean values given in the results section are sample standard deviations.

## 3 Results

### 3.1 Study part 1

This part addressed the storage ability of native cow samples (cows A,B,C,D,E,F). Blood was diluted 1:7 due to the addition of CPDA-1 solution.

#### 3.1.1 Hemograms

**Fig. 1:**
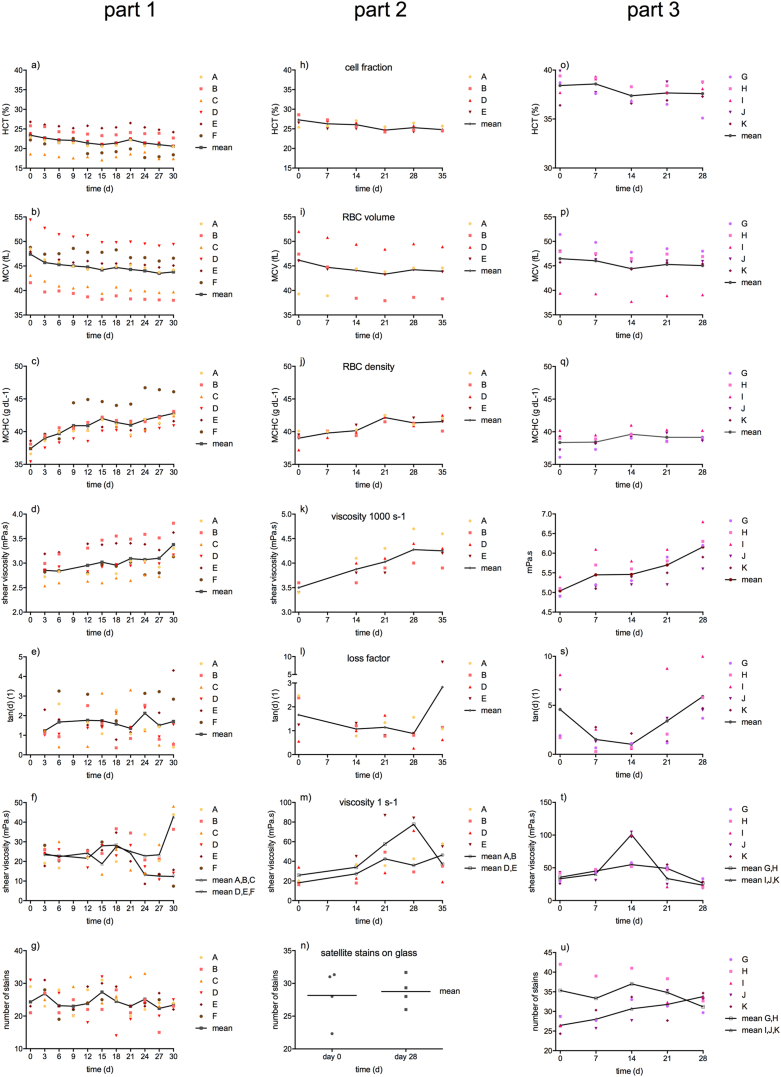
Hematological and rheological parameters, as well as number of satellite stains on glass during ageing in the three study parts.

Mean hematocrit (HCT) values decreased continuously during storage, reaching a low of 20.6 ± 2.5% on day 30 (p < 0.05; Fig. 1a), whereas the RBC counts and the mean cellular hemoglobin content (MCH) of RBCs remained stable around 5.0 T/L and 18.1 pg resp. in five of six cow blood samples (cows A-E). Continuous changes were as well observed for mean corpuscular volume (MCV) (from 47.4 ± 4.6 to 43.8 ± 4.3 fL) and for mean corpuscular hemoglobin concentration (MCHC) (from 37.4 ± 1.2 to 42.8 ± 1.8 g dL^−1^) (both p < 0.01; Fig. 1b and c). Thus, RBCs shrank with storage time, which was accompanied by an increase of cytoplasmic density. There was no hemolysis during the entire test period, except quite early in one cow sample (cow F). But surprisingly, the intensive red colour of the plasma (from day 9 onwards) was not reflected by a simultaneous decay of RBCs. In contrast, RBCs were still intact, but MCV, MCH, and MCHC were elevated. MCH increased by 3 pg per cell and remained at this level. On our next test three days later the RBC count had dropped from 4.7 to 3.9 T/L in this cow sample, which reduced HCT by 4%.

The mean number of white blood cells decreased constantly from 6.0 ± 1.2 to 3.4 ± 1.1 G L^−1^, as did the mean number of platelets (PLT) from 259 ± 92 to 177 ± 59 G L^−1^ in cows A-E (p < 0.01). There was a sudden rise of the PLT count in the hemolytic sample at day 12, which must be attributed to fragments of other cells (obviously RBCs) that were inadvertently counted as platelets.

#### 3.1.2 Rheometry

**Fig. 2:**
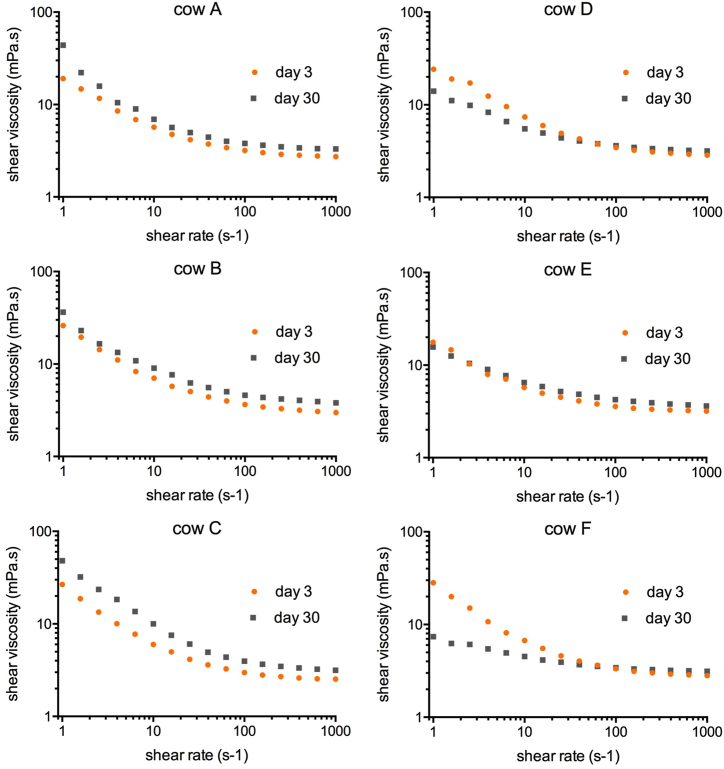
Shear thinning in study part 1. Half of the cow samples became more viscous with time (left column), whereas the other half of samples switched towards Newtonian behavior (right column). Cow F became hemolytic and displayed the highest deterioration.

High shear rate viscosity (at 1000 s^−1^) increased steadily in all samples resulting in mean values of 2.85 ± 0.22 mPa*s at the day of sampling and 3.36 ± 0.29 mPa*s on day 30 (p < 0.001; Fig. 1d). Low shear rate viscosity (at 1 s^−1^) did not show a uniform course with storage time. In fact, values varied individually around a constant mean value. The course and variability of tanD values was similar. Surprisingly, on day 27, low shear rate viscosity and tanD began to split. Samples with smaller RBCs (cows A-C) featured an increase in viscosity and a decrease of tanD, whereas in samples with larger RBCs (cows D-F) the viscosity value decreased and tanD increased (Fig. 1e and f). Consequently, the shear thinning property also split. Half of the samples tended towards Newtonian behavior (shear independent viscosity), while the other half did not (Fig. 2).

#### 3.1.3 Drip patterns

There was no change in the numbers of satellite spatter on glass with storage time (Fig. 1g). Also the size of the parent stains did not change (not shown, data can be accessed on request).

**Fig. 3:**
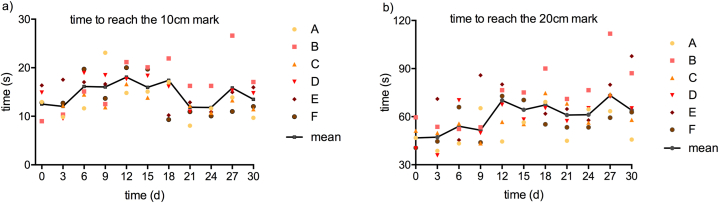
Blood on tilted glass in study part 1. The y-axis shows the time until a blood drop running down 45° tilted glass reached the given marks of 10 cm (a) and 20 cm (b).

The time it took for a blood drop to reach a 20 cm mark on a sloping glass surface increased with storage time by 27% (p < 0.05) (Fig. 3b). Values correlated well with high shear viscosity (at 1000 s^−1^) (Pearson's r = 0.56; p < 0.001), but not with values obtained at low shear conditions, both at steady, and at transient flow (viscosity at 1 s^−1^ or tanD). There was no change of time to reach the 10 cm mark (Fig. 3a).

### 3.2 Study part 2

This part served to validate study part 1. Tests were performed 4 months later by using the same animals (A,B,D,E; two individuals were no more available at Vetfarm). Samples were adjusted to a physiological bovine HCT to abolish the dilution effect through CPDA-1.

Generally, all hematological, most rheological features, and the drip pattern results on glass could be validated, although with different absolute values for several parameters (e.g., RBC count, viscosity, etc.) since HCT was changed (Fig. 1h-n). High shear rate viscosity showed an identical rise (p < 0.01), but there were differences albeit also similarities in the low shear behaviour (viscosity at 1 s^−1^ and tanD). A difference was the gain of low shear rate viscosity in part 2, since there was no such viscosity increase in part 1. A similarity was the individual behaviour of the samples thereafter. In both study parts, samples from cows A and B gained viscosity whereas samples from cows D and E switched to Newtonian behaviour. It appears that those samples that stiffen more at an earlier phase also switch earlier towards Newtonian behaviour, whereas those that do not stiffen so much are better stabilized. Only minor hemolysis was observed in one sample at day 28 (cow A). Stain size on cardstock paper did not change in aged samples compared to fresh samples (not shown, data can be accessed on request).

### 3.3 Study part 3

This part included samples with adjusted HCT (human standard). New cows were recruited (G,H,I,J,K).

#### 3.3.1 Hemograms

**Fig. 4:**
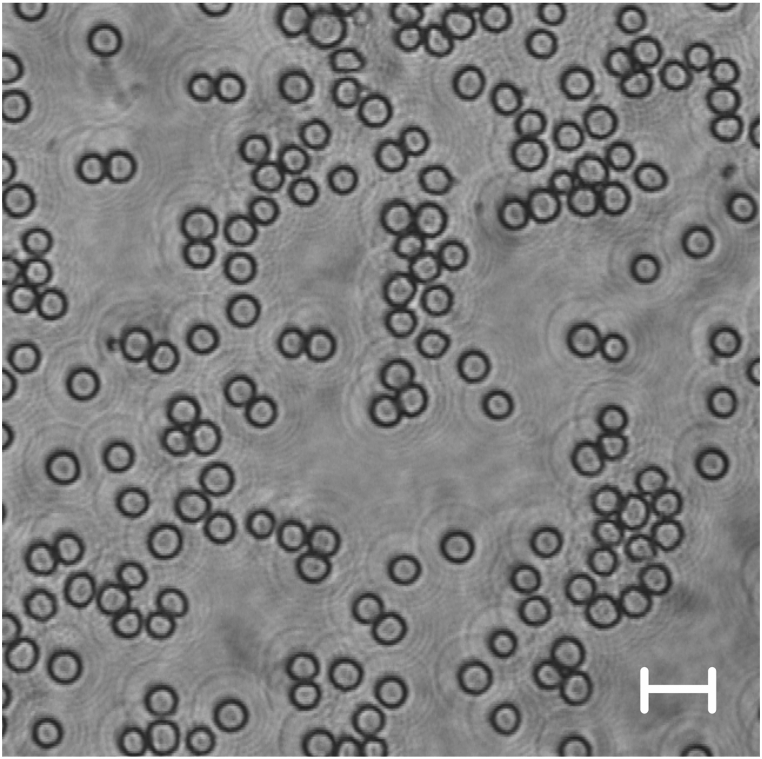
Cow RBCs in study part 3 in bright field microscopy. Erythrocytes (exemplary from cow K) still maintained round shapes on day 28. The bar indicates 10 μm.

We saw the same development of parameters like in the study parts before, but not always with the identical statistical strength (Fig. 1o-u). MCV decreased (from 46.5 ± 4.0 to 45.0 ± 3.1 fL; p < 0.05), whereas HCT and MCHC values were rather maintained (HCT decreased from 38.4 ± 1.2 to 37.6 ± 1.3%; MCHC increased from 38.3 ± 1.5 to 39.1 ± 0.5 g dL^−1^; both n.s.). There was a correlation between MCHC and MCV (r = −0.54) showing that RBCs became dense when they shrank. There was also a correlation between MCHC and high shear rate viscosity (r = 0.51, both p < 0.01) showing that the increase of cytoplasmic density paralleled the increase of blood viscosity. RBC count remained stable at 8.4 ± 0.7 T/L with RBCs maintaining their round shape like in the previous study parts (Fig. 4). MCH was stabile around 17.7 pg. PLT count decreased little (from 443 ± 83 to 374 ± 38 G/L; n.s.) but WBC count decreased severely (from 9.6 ± 1.9 to 6.6 ± 0.6 M/L; p < 0.01). Only in one sample (cow G) the plasma colour turned slightly reddish at end of storage, indicating the onset of hemolysis. No such change in colour was seen in the other samples.

#### 3.3.2 Rheology

Like in the preceding study parts, blood viscosity at high shear rate (1000 s^−1^) increased continuously (from 5.0 ± 0.2 to 6.2 ± 0.4 mPa s; p < 0.001; Fig. 1r). Blood viscosity at low shear rate (1 s^−1^) showed a biphasic course (Fig. 1t). It increased on day 14 and decreased thereafter (p < 0.01). In three animals (I,J,K) this increase was pronounced, whereas it was less dramatic in the two other samples (G,H). TanD values correlated well with low shear rate blood viscosity (r = 0.73, p < 0.005) and thus showed the opposite course (Fig. 1s). Shear thinning declined from 6.8 ± 1.4 to 4.0 ± 0.9 (p < 0.05). The maximum of low shear viscosity as well as the minimum of tanD occurred at day 14. This indicates that the shift to Newtonian behavior had started in all samples before 21 days of storage with values on day 21 becoming as low as on day 0.

#### 3.3.3 Drip patterns

**Fig. 5:**
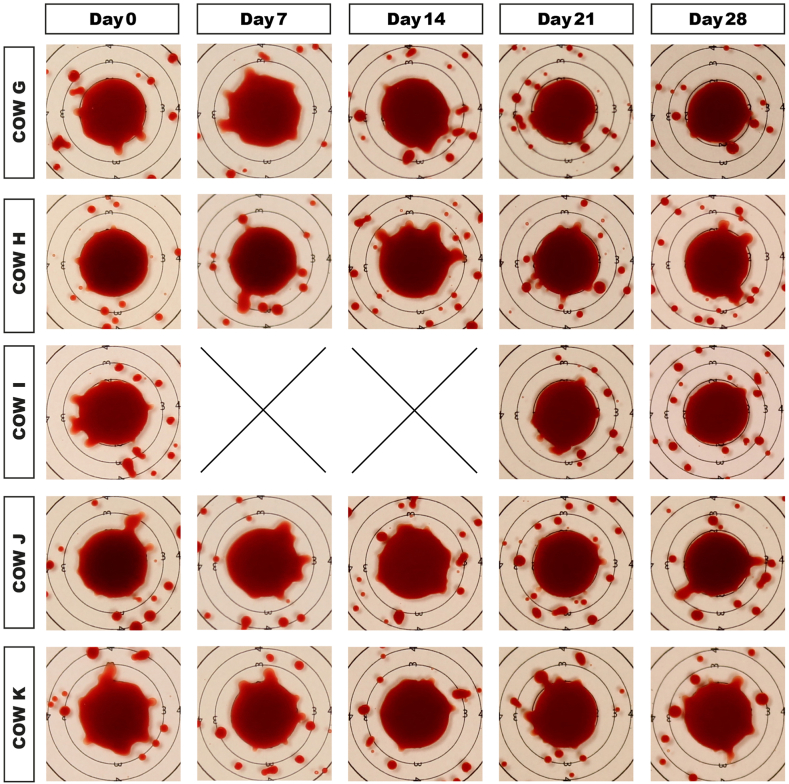
Passive drip patterns on glass in study part 3. Stain sizes became successively smaller after 14 days of storage. Due to low sample volume, two time points (days 7 and 14) could not be probed in cow I. These lacking data points are indicated by “X”.

**Fig. 6:**
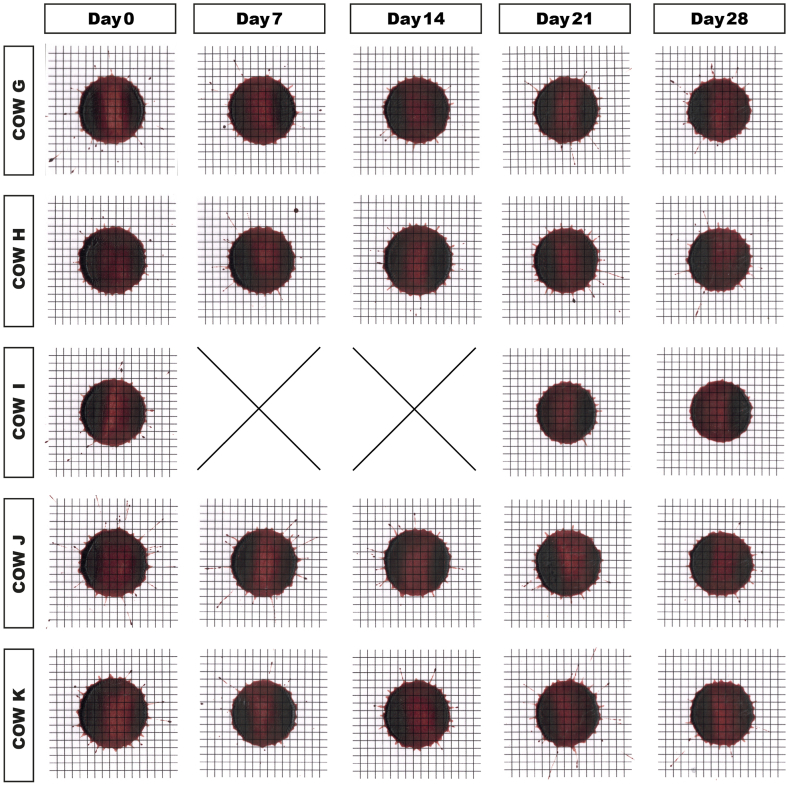
Passive drip stains on cardstock paper in study part 3. Stain sizes decreased by 7% on day 28. Due to low sample volume, two time points (days 7 and 14) could not be probed in cow I. These lacking data points are indicated by “X”.

Parent stain size on glass decreased minimally in all samples (Fig. 5). The number of satellite stains increased in three cow samples (I,J,K), decreased in one (H), and did not change in one (G) (Fig. 1u). Excluding sample H, the number of satellites correlated with the high shear viscosity (at 1000 s^−1^) over storage duration (r = 0.49, p < 0.05). It must be noted that the three cow samples that showed the dramatic low shear rate viscosity rise on day 14 (I,J,K) also displayed the highest increase in the number of satellite stains. Video 1 shows that the backsplash of blood from the point of impact was completed faster at day 28. On cardstock paper, stain size decreased in all cows with storage duration (Fig. 6) by an average value of 7%. We found a variability of stain diameters within the technical replicates of maximum 0.5 mm. Two out of the three replicates in most cases showed identical diameters.

### 3.4 Summary of results obtained in all study parts

Similar findings: HCT decreased during storage - not due to a decay of RBCs, but due to a decrease of MCV. PLT and WBC counts decreased. MCHC increased together with high shear rate blood viscosity. RBCs maintained round shapes and hemolysis was minimal.

Different findings were seen in rheology at low shear condition (viscosity at 1 s^−1^ and tanD) and in passive drip patterns. In study part 1, viscosity and tanD were maintained long-term (varying around a mean value) and only switched clearly into a certain direction after 24 days of storage: blood samples separated into those that became more fluidic, and another half of samples that became more condensed (Fig. 2). In study part 2, viscosity was not maintained but increased sucessively with storage duration. While viscosity increased further in two cows, it displayed a maximum in two other cows at 28 days of storage. Blood from the same cows as in part 1 switched towards Newtonian behavior. In study part 3 this low shear viscosity maximum already occurred at 14 days of storage, and all five cows tended towards Newtonian behavior thereafter. Samples whose viscosity peaked at higher values got closer to Newtonian behavior than samples that only little increased their viscosity at 14 day. Stain sizes and the number of satellite spatter around the point of impact did not change when HCT was low. Only when HCT was elevated to a value physiological to human, the characteristic decrease of stain size observed elsewhere [6,9] was detected, and satellites became numerous. The development of suspension property with storage time parallels the number of satellite stains on glass. Those samples that deteriorated most showed the highest change in the number of satellite spatter.

## 4 Discussion

Cow RBCs remained intact throughout 30 days of storage in all study parts, which makes cow blood a more stable option than swine blood drawn and stored under identical conditions since all pig samples became hemolytic [6]. As a sign of ageing [10], cow RBCs shrank, but only very few cells took on irregular shapes. The maintenance of the cell shape is remarkable in terms of the pronounced tendency of other species' RBCs to form echinocytes or spherocytes when they are stored [11,12]. Eryptosis is thus delayed in stored cow RBCs [13]. One exception to this is the cow sample that became hemolytic at an already early stage of storage in study part 1. In this hemolytic sample, several spiculated and teardrop shaped RBCs were present showing severe cell damage. Expectedly the RBC count dropped by one sixth. The onset of minimal hemolysis in one sample of part 3 was not accompanied by the presence of irregular RBC shapes.

RBC shrinking without a simultaneous change into irreversible or apoptotic shapes implies cellular mechanisms that maintain the symmetric resting shape. Cow RBCs have been defined as relatively stiff on whole cell level (see table 12.2 in Ref. [3]), which assumes a stabilized membrane. On membrane level, not much is known about cow RBCs. Generally stiffness is related to the cytoskeleton mechanics and to the quality of the anchor points between the spectrin meshwork and the bilayer. One of these anchor points is the actin – protein-4.1R complex that loses its connection to the bilayer upon phosphorylation [14,15]. When intracellular ATP is consumed during storage, cell stiffening is a logical consequence. Another anchor point comprises the band 3 protein – ankyrin complex [16]. In fresh RBCs, band 3 is present as dimers (80% of the membrane protein content used for anion exchange) and as tetramers (20% of the content used for membrane stabilisation) [17]. During storage band 3 successively oligomerizes [18], which changes this quantitative relationship. The transmembrane domains of oligomers have a lower rotational mobility [19] and therefore contribute to membrane stiffness. The clustering of band 3 molecules in the bilayer - with and without assembly with other membrane proteins [20] - must mean that the membrane area between these protein complexes increases. This facilitates membrane shedding during ageing [18,20]. The reduced membrane area makes cells smaller and rounder, but only if all membrane components are equally distributed over the cell surface. Otherwise, the shape will be irregular. In cows, the higher stability of band 3 clusters [21] and the lower cohesion of membrane phospholipids [22] might thus favor the round cell shape observed here. Another difference to pig blood is the lack of RBC swelling, which usually follows intracellular ATP depletion. This is surprising, because bovine RBCs are very sensitive to fluctuations in osmotic pressure [23]. Since our shortest interval for analysis was three days, a swelling - if present - must have occurred within the first two days and was therefore only transient. The lack of aquaporin-3 in cow RBC membranes [24] might explain this finding. As only RBC size, but not RBC count changed with time, MCH remained constant, but HCT steadily decreased with storage.

A decrease in HCT usually lowers blood viscosity. But viscosity (at 1000 s^−1^) increased continuously from start to end in all samples. Such an increase in viscosity has already been observed in earlier ex-vivo animal studies [6,9] being a common feature of blood storage [25]. It can be explained by the progressive reduction of RBC flexibility due to several oxidative and metabolic alterations summed up as “storage lesion”. Lipid peroxidation, protein oxidation (including hemoglobin), loss of ATP and nitric oxide bioactivity, lactate and ROS accumulation, 2,3-DPG depletion, loss of peroxidase activity, carbonylation and glycation are prominent mechanisms that damage structural components and make the RBC surface rough and porous. Nanovesiculation followed by their release into the surrounding makes RBCs additionally stiff due to the loss of membrane area [20,[26], [27], [28], [29], [30]]. The rise in cytoplasmic density with storage duration [31], measured by the MCHC index in this study, as well as the surge of membrane stiffness as explained above increase the flow resistance of RBCs and cannot be balanced by the simultaneous HCT reduction. This is reflected in the decelerated flow of blood drops along an inclined surface, shown in study part 1.

The end of shelf life is certainly marked by the shift towards Newtonian behavior and identifies the point at which the suspension becomes unstable due to the manifold effects on blood cells. This shift to Newtonian behavior occurs after a period of increasing elasticity. This time point can be assessed through flow curves at a wide range of shear rates. Shear rates higher than 100 s^−1^ alone will not provide this information [25]. Newtonian behavior (shear-independent viscosity) is observed if the components of a sample do not interact [32]. Translated to a blood suspension, this means that blood components – individual blood cells, protein clusters, and/or cell clusters – form separate units, which lack a collective behavior. Such a change in behavior with storage time can result from altered surface properties of the suspended units [33,34] and from steric factors that affect the fluid flow between them. Unstable local shear rates for example occur, when large, stiff clusters tumble and disrupt the attempts of others (smaller, softer units) to get into more coordinated motions. The result is a reduction of shear thinning, which we observed in part 3 in all samples, and in parts 1 and 2 only in samples with larger RBCs. The timely onset of this shift towards Newtonian behavior depends on the history of the samples stored and their HCT. This asks for an explanation. We observed that samples that became significantly condensed with time (all data points on the viscosity curve shifted upwards) also fluidified more abruptly thereafter (viscosity curve tended to horizontal). Samples where low shear rate viscosity was only half as large (compare with Fig. 1t) did not deteriorate that much afterwards. Therefore, if low shear blood viscosity values suddenly get high, one can expect that they will shortly drop. This means that the suspension dynamics change within few days from becoming more shear thinning to becoming less shear thinning. Such a shift was also seen in ageing pig blood. When it takes place, one has to reckon with altered bloodstain patterns. In contrast, patterns were less altered when the rheological changes were less dramatic (compare with Fig. 1u). We observed that these behavioural changes occurred earlier at higher HCT values. All functional and morphological alterations have a greater impact on the suspension quality when the HCT is high. This is because the RBCs (obviously the number and not the size) add structural complexity to the system. If samples are diluted through CPDA-1 not only the HCT is reduced, but also the plasma protein concentration, which supports the fluidic state. But just as well, the lower steric stabilisation of clusters in diluted samples might be a problem. In samples with low HCT the quasi-static behavior (Fig. 1e) appeared incidental, showing high fluctuations around a mean value until storage day 24. Generally, tests at small oscillating shear amplitudes (in linear mode) are very sensitive to the presence of structures that span the gap. When the shape and/or the stiffness of structures change, the shear moduli change in parallel. In diluted samples the clusters are better moveable and a potential condensation may not be traceable earlier because fewer clusters do not always span the gap. We also think it is important to mention that the low RBC aggregability of bovine RBCs [4] and the low yield stress of freshly drawn cow blood [35] does not protect the samples from becoming more elastic (= gel-like) at a certain time point during storage. The increase in viscosity and elasticity was also observed in human blood stored under blood bank conditions [36], and might be a general feature during storage. But it comes before the blood shifts toward the Newtonian behavior.

Regarding the maximum storage time of cow blood, we conclude that it depends on the samples' HCT. If the HCT is adjusted to a human physiological value, cow samples should not be older than 14 days. If native samples are used, they can be used one more week. Fig. 1e, L, and 1s show that the minimum for tanD occurs between 14 and 28 days in the different study parts. Within this range, the samples are no more in their native condition, but do not yet shift in the direction of Newtonian behavior, which we define as the endpoint for storage. This shelf life is of course related to the conditions that we set: drawing blood from conscious animals in their familiar environment, using an anticoagulant like CPDA-1 that supports RBC viability, storing the samples at refrigerator temperatures, and mixing them smoothly every third day. Samples stored under other conditions will have other shelf lifes.

What is necessary to emphasize is the fact that the timely onset of hemolysis occurs later than the deterioration of suspension quality. This is different to pig blood [6] and must be based on the stabile bovine RBC membrane including its cytoskeletal attachment [21,22]. Clear plasma of a stored cow sample does therefore not indicate unaltered suspension property. Likewise, the viscosity value at high shear rate that is easier to measure than its counterpart at low shear rate is alone unsuitable for a qualitative estimation. Interesting is also the fact that the stain sizes changed only slightly, both on glass, and on cardstock paper (diameter reduced by only 7%), which is in contrast to stored pig blood [6,37]. Only the increase of satellite stains around the point of impact was significant with ageing. Video 1 shows that the blood splashing backwards was reduced, like with a coil that looses its springiness (cf. Maxwell model [38]). This loss of elasticity is supported by the increase of tanD (Fig. 1s). We conclude that relevant changes occur in cow samples upon storage, even if RBC functionality parameters [39,40] or stain sizes give only little evidence of this. But the reduction of quality is not as apparent as in pigs.

The reader may allow an additional note to be adressed as to the choice of the animal blood in BPA. The preservation of the round RBC shape, the lack of RBC swelling, the clear limitation of hemolysis, and the minimal reduction in stain size on different substrates indicates cows as a better blood source than pigs, especially since cow blood is also easier to draw outside slaughterhouses. The lower shelf life of cow samples (14 days based on blood rheology) compared to pig samples (21 days based on rheology and hemolysis) when HCT is adjusted to a human standard is however troubling. It could result from non-physiological cell crowding eliciting mechanisms not predominant at physiological HCT. Nevertheless, the native bovine HCT is too low and must be adjusted so that it reflects a victim's value. For the adjustment it must be noted that a 10% increase in HCT in a bovine sample will not produce the same level of viscosity of a 10% increase in HCT in a human sample, since RBC properties and the HCT dependence of blood viscosity are species-specific features. Any HCT adjustment of animal blood samples must therefore be done with the simultaneous consideration that blood viscosity changes with different gradients, also influenced by temperature [1]. One must decide which parameter (HCT or viscosity) is most relevant and should thus be set to the human standard.

## Declarations

### Author contribution statement

Ursula Windberger: Conceived and designed the experiments; Performed the experiments; Analyzed and interpreted the data; Contributed reagents, materials, analysis tools or data; Wrote the paper. Andreas Sparer: Performed the experiments; Analyzed and interpreted the data; Wrote the paper. Johann Huber: Performed the experiments; Contributed reagents, materials, analysis tools or data.

### Funding statement

This research did not receive any specific grant from funding agencies in the public, commercial, or not-for-profit sectors.

### Data availability statement

Data associated with this study has been deposited at https://hal.archives-ouvertes.fr/hal-03768526.

### Declaration of interest’s statement

The authors declare no conflict of interest
